# Shared and condition-associated gut microbiota alterations in older adults with depression and constipation: evidence from the American Gut Project

**DOI:** 10.3389/fmicb.2026.1891231

**Published:** 2026-07-08

**Authors:** Xiang Li, Leyong Ke, Ting Wang, Ziqiu Lei, Feng Tian, Yan Zhang, Xin Liu

**Affiliations:** 1 Department of Gastroenterology, Zigong Fourth People's Hospital, Zigong, China; 2 Department of Plastic Surgery, Zigong Fourth People's Hospital, Zigong, China; 3 Department of Gastroenterology, The Affiliated Hospital of Southwest Medical University, Luzhou, China; 4 Department of Clinical Laboratory, Zigong Fourth People's Hospital, Zigong, China

**Keywords:** 16srRNA, American Gut Project, constipation, depression, microbiota, older adults

## Abstract

**Background:**

Constipation and depression frequently co-occur in older adults, and growing evidence suggests that gut microbiota dysbiosis may be a shared feature of both conditions. The microbiota has well-established roles in gastrointestinal motility and gut–brain axis signaling, and compositional alterations have been independently reported in each condition. However, whether older adults with constipation and those with depression share common microbiota characteristics have not been systematically investigated.

**Aim:**

This study aimed to characterize gut microbiota alterations in older adults with depression or constipation using 16S rRNA amplicon sequencing data from the American Gut Project, focusing on microbial features shared by, or specific to, the two conditions.

**Methods:**

We retrieved fecal 16S rRNA sequencing data from 513 older adults in the publicly available American Gut Project database, including HC (*n* = 277), DP (*n* = 78), and CP (*n* = 158). We compared alpha and beta diversity, taxonomic composition, and genus-level differential abundance among groups, used random forest models to explore features contributing to group discrimination, and performed covariate-adjusted and sensitivity analyses to assess robustness.

**Results:**

Alpha diversity was comparable among groups, whereas beta diversity revealed detectable differences in community composition. After adjustment for age, sex, and BMI, Bray-Curtis-based differences remained evident, with the most consistent pairwise difference between CP and HC. At the genus level, CP showed depletion of health-associated butyrate-producing taxa and enrichment of selected mucin- or inflammation-associated taxa, whereas DP was characterized by enrichment of Erysipelatoclostridium and [Ruminococcus]_gnavus_group and depletion of UCG-002 and selected health-associated genera. Random forest analyses further identified key microbial contributors to group discrimination.

**Conclusion:**

We identified subtle and partially overlapping genus-level microbiota alterations in older adults with constipation and depression, with constipation showing the most consistent differences from healthy controls. These findings provide exploratory evidence that selected microbiota alterations may be relevant to the clinical overlap between the two conditions, although their functional roles require validation in longitudinal studies integrating metagenomic and metabolomic profiling.

## 1 Introduction

Constipation and depression are among the most prevalent conditions affecting older adults, imposing a substantial burden on both individual quality of life and public health systems. Constipation is characterized by difficult passage of stool, prolonged intestinal transit time, reduced defecation frequency, and a sensation of incomplete evacuation. Globally, chronic constipation affects approximately 14% of the general population (Suares and Ford, 2011), rising to 33% among individuals over 60 years of age (Lee et al., 2025). Regional surveys have reported considerable variation in prevalence across Asian countries, ranging from 2.0% in Indonesia to 16.7% in the Philippines (Ren et al., 2025). The burden of chronic constipation in older adults is particularly concerning given its strong association with psychiatric comorbidities. Survey data indicate that 50.82% of older patients with constipation were diagnosed with anxiety or depression, and only 19.20% were free from anxiety symptoms (Liang et al., 2022). Depression has been increasingly recognized as a common complication of chronic constipation in older adults, a relationship confirmed by numerous clinical studies (Bharucha et al., 2013).

The gut microbiota represents a vast and complex ecosystem of approximately 100 trillion microorganisms (Berg et al., 2020), performing essential functions including digestion, preservation of intestinal homeostasis (Fujisaka et al., 2023), immune regulation, and maintenance of the intestinal mucosal barrier (Chu and Mazmanian, 2013). Growing evidence links both constipation and depression to gut microbiota dysbiosis. In constipation patients, the abundances of *Bacteroides, Roseburia*, and *Faecalicoccus* are decreased, while individuals with depression show altered levels of *Peptoniphilus, Pseudoramibacter-Eubacterium*, and *Candidatus-Solibacter* (Liang et al., 2022). Furthermore, *Aquicella* and *Limnohabitans* have been negatively correlated with both constipation symptoms and depression. However, whether older adults with constipation and those with depression share common gut microbiota characteristics remains to be systematically investigated.

In this study, we analyzed fecal microbiota composition in older adults with constipation, depression, and healthy controls using 16S rRNA amplicon sequencing data from the American Gut Project. We aimed to characterize shared and condition-associated gut microbiota alterations in constipation and depression, with the goal of determining whether these two clinically associated conditions show overlapping microbial patterns in older adults.

## 2 Methods

### 2.1 Study subjects & clinical data collection

We retrieved fecal microbial sequencing data from the Gut Microbiota Repository (GMrepo; https://gmrepo.humangut.info) (Liu et al., 2026), a curated database of consistently annotated human gut metagenomes. We downloaded raw sequencing data from the NCBI Sequence Read Archive (SRA) using sample identification numbers obtained from GMrepo.

All samples were derived from the American Gut Project (AGP), the world's largest citizen science initiative for human gut microbiota research, which has profiled over 10,000 samples using both 16S rRNA sequencing and shotgun metagenomic approaches. We obtained phenotype labels and sample identifiers from GMrepo and included only samples originating from the American Gut Project (project ID: PRJEB11419). The following inclusion criteria were applied: (1) phenotype classified as depression, constipation, or healthy control; (2) participant age ≥60 years; (3) fecal samples sequenced by 16S rRNA gene amplicon sequencing targeting the V4 region; and (4) samples passing quality-control criteria. Participants meeting criteria for both depression and constipation were excluded from the primary analysis to maintain mutually exclusive group assignment. Phenotype classification was based on the available GMrepo metadata rather than clinician-adjudicated diagnoses. Demographic and anthropometric variables, including age, sex, and BMI, were extracted for baseline description and covariate-adjusted analyses.

### 2.2 16S rRNA gene sequencing analysis

We processed all raw sequencing data using QIIME 2 (Quantitative Insights Into Microbial Ecology), performing quality filtering and chimera removal to retain high-quality sequences. We then denoised the sequences into Amplicon Sequence Variants (ASVs) and annotated them taxonomically using the SILVA reference database (version 138). Downstream statistical analyses of ASV tables were conducted using the STAMP software package (version 2.1.3).

We calculated alpha and beta diversity using QIIME 2. We calculated alpha diversity using four indices: the Shannon diversity index, Faith's phylogenetic diversity index, Pielou's evenness index, and Simpson's index, collectively capturing microbial richness and community evenness within each sample. For beta diversity, we used Bray-Curtis dissimilarity and visualized the results through Principal Coordinate Analysis (PCoA). We applied PERMANOVA to test for significant differences in community structure among groups, and ANOSIM based on Bray-Curtis distance to determine whether inter-group variation exceeded intra-group variation. We employed Linear Discriminant Analysis Effect Size (LEfSe) to identify differentially abundant taxa between groups, using *P* < 0.05 and LDA score > 3.5 as commonly used, relatively stringent thresholds for identifying taxa with stronger discriminatory contributions in microbiome LEfSe analyses (Deng et al., 2022; Yuan et al., 2025).

### 2.3 Statistical analysis

We performed statistical analyses using R (version 4.2.2) and Python. Continuous variables are presented as mean ± standard deviation (SD) and were compared among groups using ANOVA, whereas categorical variables are expressed as frequencies and percentages and were analyzed using chi-square tests. We compared genus-level microbial abundances among groups and further evaluated key associations using models adjusted for age, sex, and BMI. We controlled multiple testing using Benjamini-Hochberg false discovery rate (FDR) correction across tested genera within each pairwise comparison, considering *q* < 0.05 statistically significant. We reported associations with nominal *P* < 0.05 but *q* ≥ 0.05 as exploratory findings. Based on prior microbiome literature and taxa highlighted by the initial differential abundance and LEfSe analyses, we further examined candidate genera in DP vs. HC comparisons, including *Agathobacter, Faecalibacterium, Akkermansia, Prevotella*, UCG-002, UCG-005, *Roseburia, Bacteroides, Erysipelatoclostridium*, [*Ruminococcus*]_gnavus_group, *Flavonifractor, Coprococcus*, and *Holdemanella*. We constructed pairwise random forest models to evaluate discriminative performance for CP vs. HC, DP vs. HC, and CP vs. DP using stratified 5-fold cross-validation, receiver operating characteristic (ROC) curves, and area under the curve (AUC). In parallel, we constructed a three-class random forest model incorporating genus-level microbial abundance features, age, sex, and BMI to prioritize microbial features contributing to overall group discrimination among CP, DP, and HC.

## 3 Results

### 3.1 Clinical characteristics of participants

A total of 513 older adults were included in the final analysis, comprising 158 participants in the CP group, 78 in the DP group, and 277 in the HC group. The mean ages of the CP, DP, and HC groups were 65.71 +/– 4.37, 65.10 +/– 3.94, and 66.30 +/– 5.04 years, respectively, with no significant differences among groups (*P* = 0.108). However, sex distribution differed significantly among groups (*P* < 0.001), and BMI also differed significantly (*P* < 0.001), with the DP group showing higher BMI than the CP and HC groups. These imbalances were therefore considered in subsequent covariate-adjusted analyses. Detailed clinical characteristics of all participants are summarized in Table 1.

**Table 1:** The clinical characteristics of participants.

Characteristic	Category	CP	DP	HC	*P-value*
Age		65.71 ± 4.37	65.10 ± 3.94	66.30 ± 5.04	0.108
Gender	Female	117 (74.0%)	58(74.3%)	96 (34.7%)	< 0.001
	Male	41(26.0%)	18 (23.1%)	177 (63.9%)	< 0.001
	Unknown	0 (0.0%)	2 (2.6%)	4 (1.4%)	-
BMI		24.69 ± 4.82	27.27 ± 5.09	24.61 ± 3.88	< 0.001

### 3.2 Alpha and beta diversity analysis

**Figure 1:**
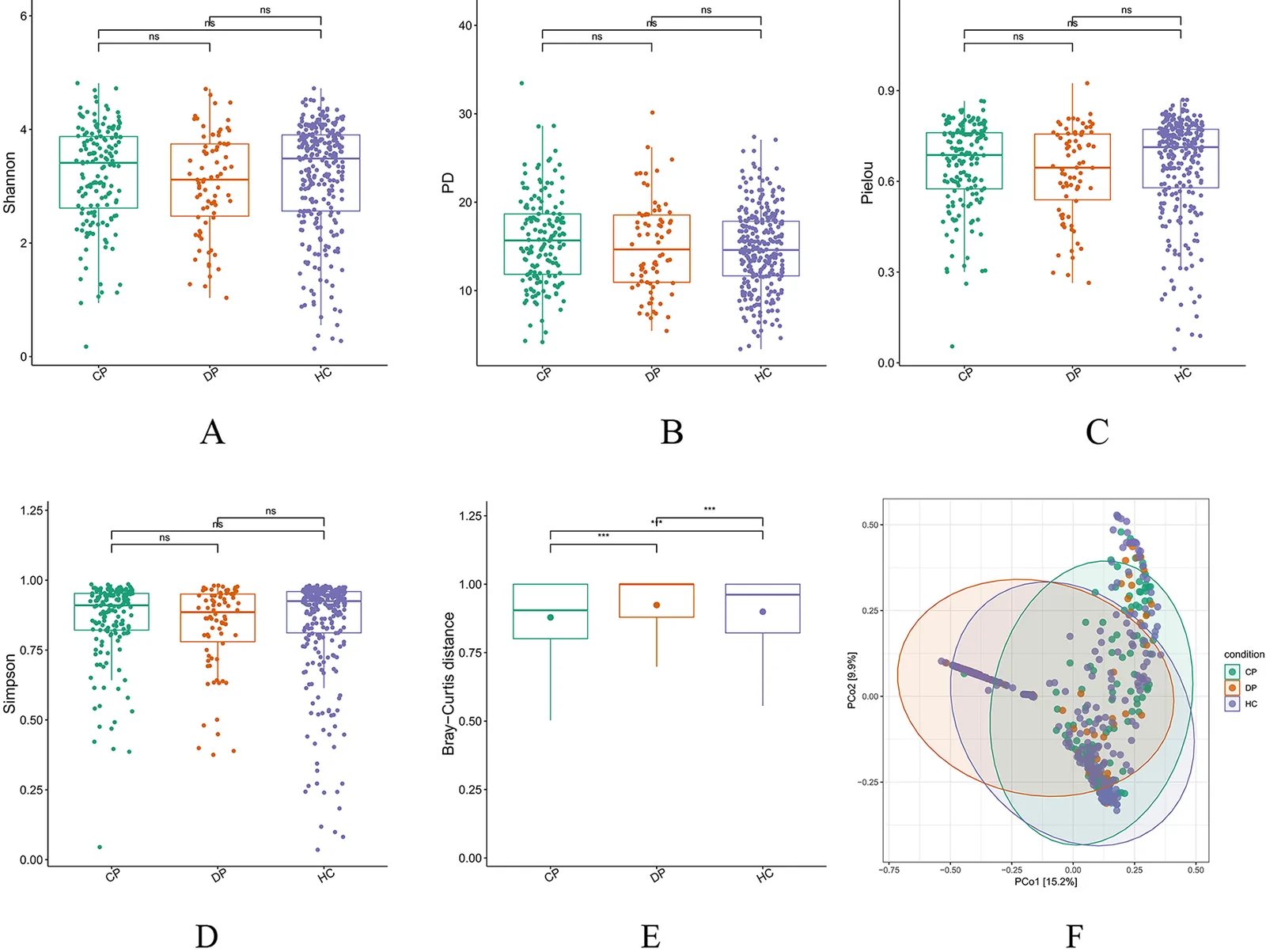
Alpha and beta diversity of gut microbiota across the CP, DP, and HC groups. **(A–D)** Alpha diversity indices, including Shannon diversity, Faith's phylogenetic diversity, Pielou's evenness, and Simpson's index, did not differ significantly among groups (all *P* > 0.05). **(E)** Bray-Curtis dissimilarity distances showing beta diversity differences among groups. **(F)** PCoA based on Bray-Curtis dissimilarity showing substantial overlap among CP, DP, and HC samples, indicating that the detected community-level differences did not correspond to clearly separated clusters (PCo1 = 15.2%, PCo2 = 9.9%). The symbols indicate statistical significance: ^***^p < 0.001; ns, not significant.

We assessed alpha diversity using four indices: the Shannon diversity index (Figure 1A), Faith's phylogenetic diversity index (Figure 1B), Pielou's evenness index (Figure 1C), and Simpson's index (Figure 1D). No significant differences were observed among the CP, DP, and HC groups across all four indices (*P* > 0.05), indicating comparable microbial richness and community evenness across groups.

Beta diversity analysis suggested differences in overall microbial community structure among the CP, DP, and HC groups. In the unadjusted Bray-Curtis analysis, pairwise differences were observed among groups (Figure 1E). After adjustment for age, sex, and BMI, the overall group effect remained statistically significant (*P* = 0.026). In pairwise adjusted analyses, the CP-HC comparison showed the clearest difference (*P* = 0.028), whereas the DP-HC (*P* = 0.417) and CP-DP (*P* = 0.109) comparisons showed more limited separation. PCoA explained 15.2% and 9.9% of the total variance along PCo1 and PCo2, respectively (Figure 1F), and showed substantial overlap among groups, indicating that the detected community-level differences did not correspond to clearly separated clusters.

### 3.3 Gut microbiota composition across the three groups

**Figure 2:**
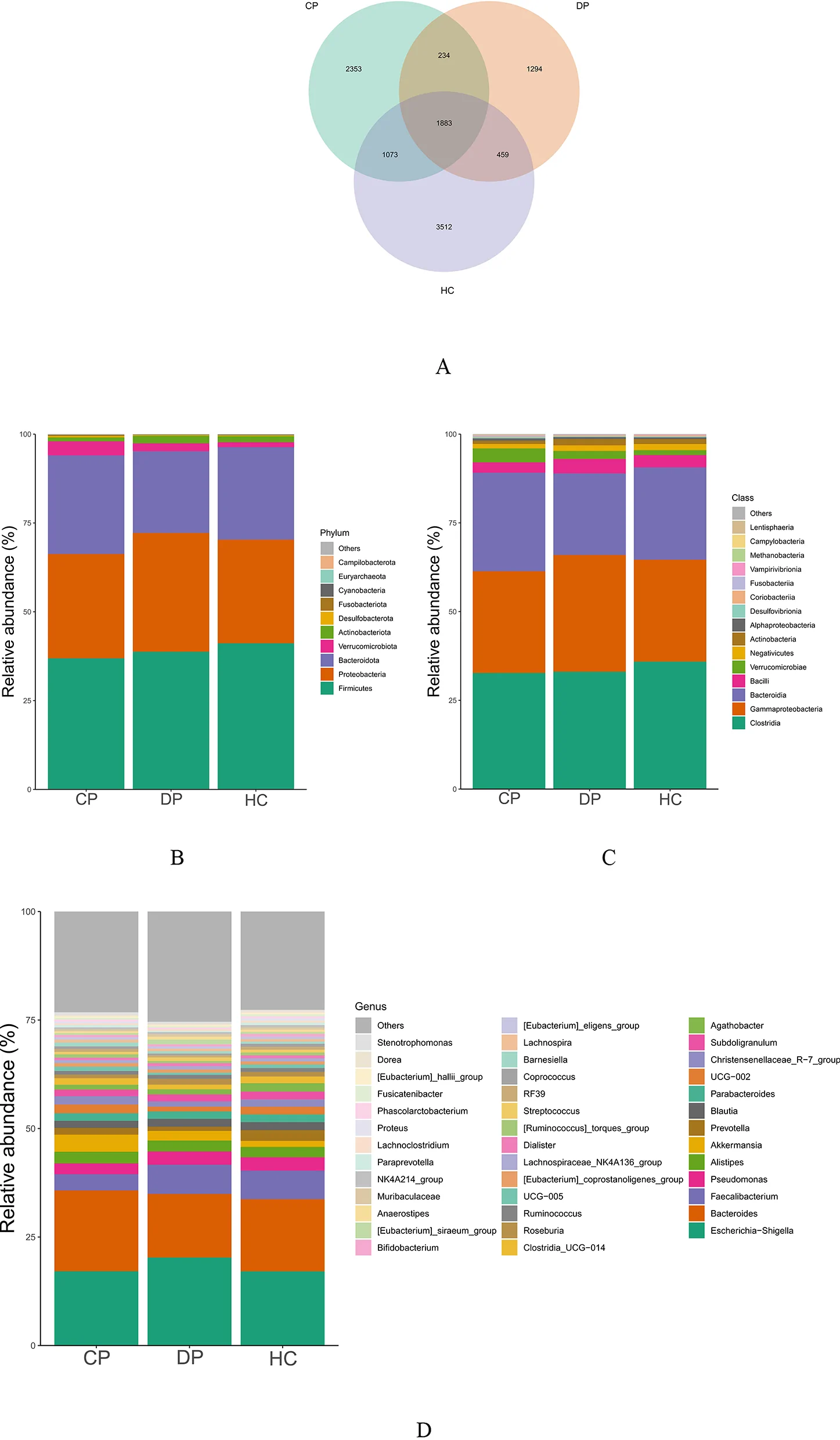
Gut microbiota composition across the three groups. **(A)** Venn diagram showing shared and unique ASVs among the CP, DP, and HC groups. **(B)** Relative abundance of the dominant phyla across the three groups. **(C)** Relative abundance of the dominant classes across the three groups. **(D)** Relative abundance profiles of the top 40 most abundant genera across the three groups.

A total of 10,808 ASVs were identified across all samples. Venn diagram analysis revealed that 1,883 ASVs were shared among all three groups, while 2,353, 1,294, and 3,512 ASVs were unique to the CP, DP, and HC groups, respectively (Figure 2A).

At the phylum level, *Firmicutes, Proteobacteria*, and *Bacteroidota* were the three dominant phyla across all groups, collectively accounting for the majority of the gut microbiota (Figure 2B). At the class level, *Clostridia, Gammaproteobacteria*, and *Bacteroidia* were the most abundant classes across all three groups (Figure 2C). At the genus level, *Escherichia-Shigella* and *Bacteroides* were the two predominant genera, collectively accounting for over 30% of the total gut microbiota. Together with *Faecalibacterium, Pseudomonas, Alistipes, Akkermansia*, and *Prevotella*, these dominant genera accounted for more than 50% of the total microbiota across all groups, with notable compositional differences observed among the three groups (Figure 2D).

### 3.4 Differential gut microbiota features among the CP, DP, and HC groups

**Figure 3:**
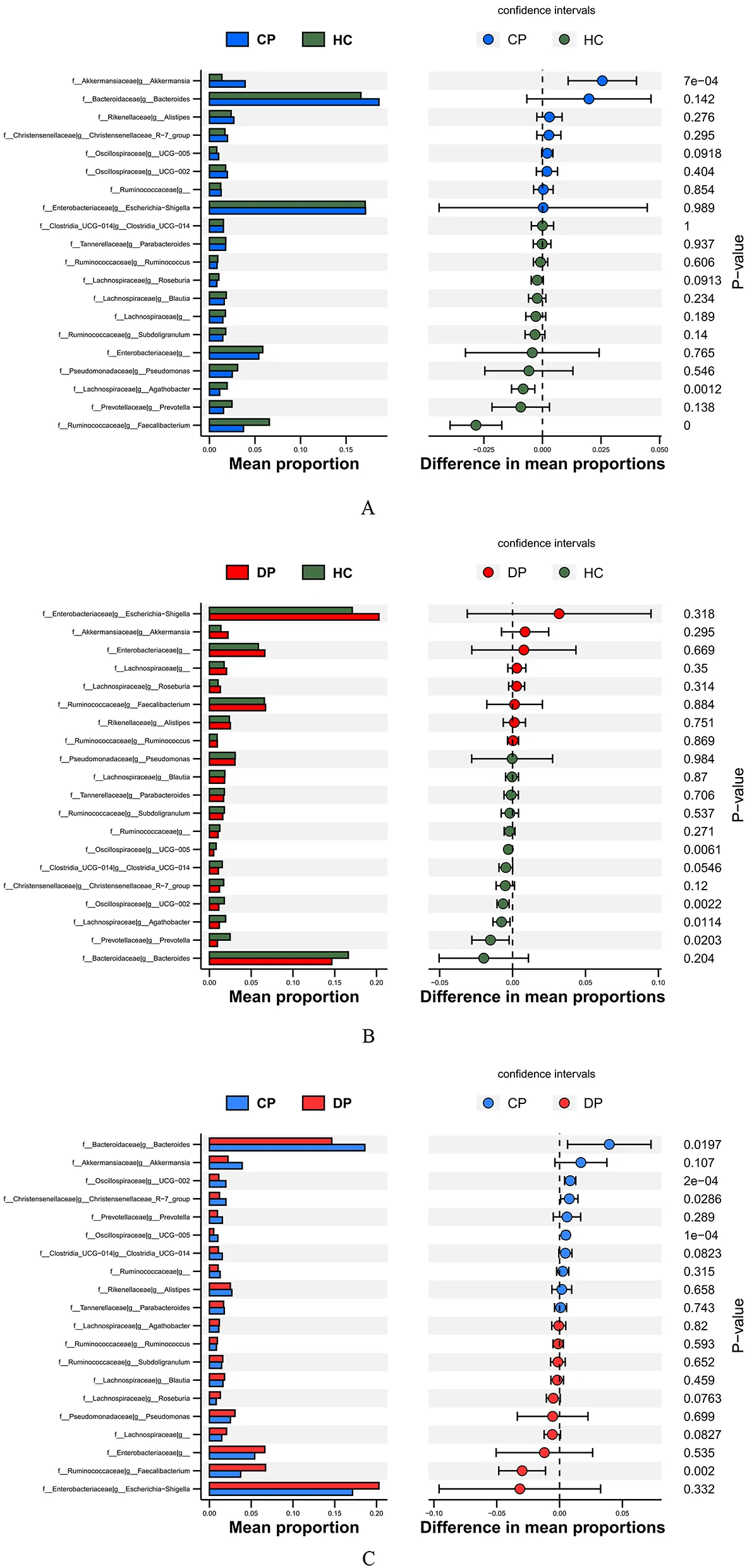
Differential abundance of the top 20 most abundant genera between groups. **(A)** CP vs. HC group. **(B)** DP vs. HC group. **(C)** CP vs. DP group.

Figure 3 shows the initial genus-level differential abundance patterns among the three groups. To evaluate the robustness of these findings, we further performed covariate-adjusted genus-level analyses controlling for age, sex, and BMI, Benjamini-Hochberg FDR correction, BMI-overlap analyses for key taxa, and DP-focused candidate-genera analyses.

Compared with HC, the CP group showed increased abundance of *Akkermansia* and reduced abundances of *Agathobacter* and *Faecalibacterium* in the initial pairwise analysis (Figure 3A). In the covariate-adjusted and FDR-corrected analyses, CP vs. HC retained multiple significant genus-level differences, including enrichment of *Akkermansia*, UBA1819, [*Clostridium*]_innocuum_group, and *Coprobacillus*, as well as depletion of *Faecalibacterium* (*q* < 0.05). BMI-overlap analysis further supported CP-associated differences in *Akkermansia, Faecalibacterium, Agathobacter*, and UCG-005. These results indicate a consistent CP-associated microbial pattern involving mucin-associated taxa, inflammation-related taxa, and health-associated butyrate-producing bacteria.

For DP vs. HC, the initial pairwise analysis showed reduced abundances of UCG-002, *Agathobacter, Prevotella*, and UCG-005 (Figure 3B). Across complementary covariate-adjusted, FDR-corrected, and DP-focused sensitivity analyses, the DP-associated pattern was characterized by depletion of UCG-002 and selected health-associated genera, together with enrichment of *Erysipelatoclostridium* and [*Ruminococcus*]_gnavus_group. These findings suggest that depression-related microbiota alterations are reflected primarily at the genus level and involve both loss of fiber- or SCFA-associated taxa and enrichment of selected dysbiosis-associated taxa.

In the initial CP vs. DP comparison, *Bacteroides*, UCG-002, Christensenellaceae R-7 group, and UCG-005 were more abundant in CP, whereas *Faecalibacterium* was more abundant in DP (Figure 3C). After covariate adjustment and FDR correction, these CP-DP differences were attenuated, indicating that the two disease groups were not strongly separated at the genus level. Together with the CP-HC and DP-HC findings, this pattern suggests that constipation and depression may share partially overlapping microbiota alterations while retaining selected condition-associated microbial features.

The key differentially abundant genera identified in the initial pairwise comparisons are summarized in Table 2. The adjusted and sensitivity analyses further refined these findings by identifying CP- and DP-associated microbial features that were more robust to baseline imbalances and multiple-testing correction.

**Table 2:** Summary of genus-level differences identified among the CP, DP, and HC groups based on STAMP analysis.

Genus	CP vs. HC	DP vs. HC	CP vs. DP
*Akkermansia*	↑	ns	ns
*Agathobacter*	↓	↓	ns
*Faecalibacterium*	↓	ns	↓
*Prevotella*	ns	↓	ns
UCG-002	ns	↓	↑
UCG-005	ns	↓	↑
*Christensenellaceae* R-7 group	ns	ns	↑

↑, significantly enriched; ↓, significantly depleted; ns, not significant (*p* > 0.05).

### 3.5 LEfSe analysis of discriminatory microbial taxa

**Figure 4:**
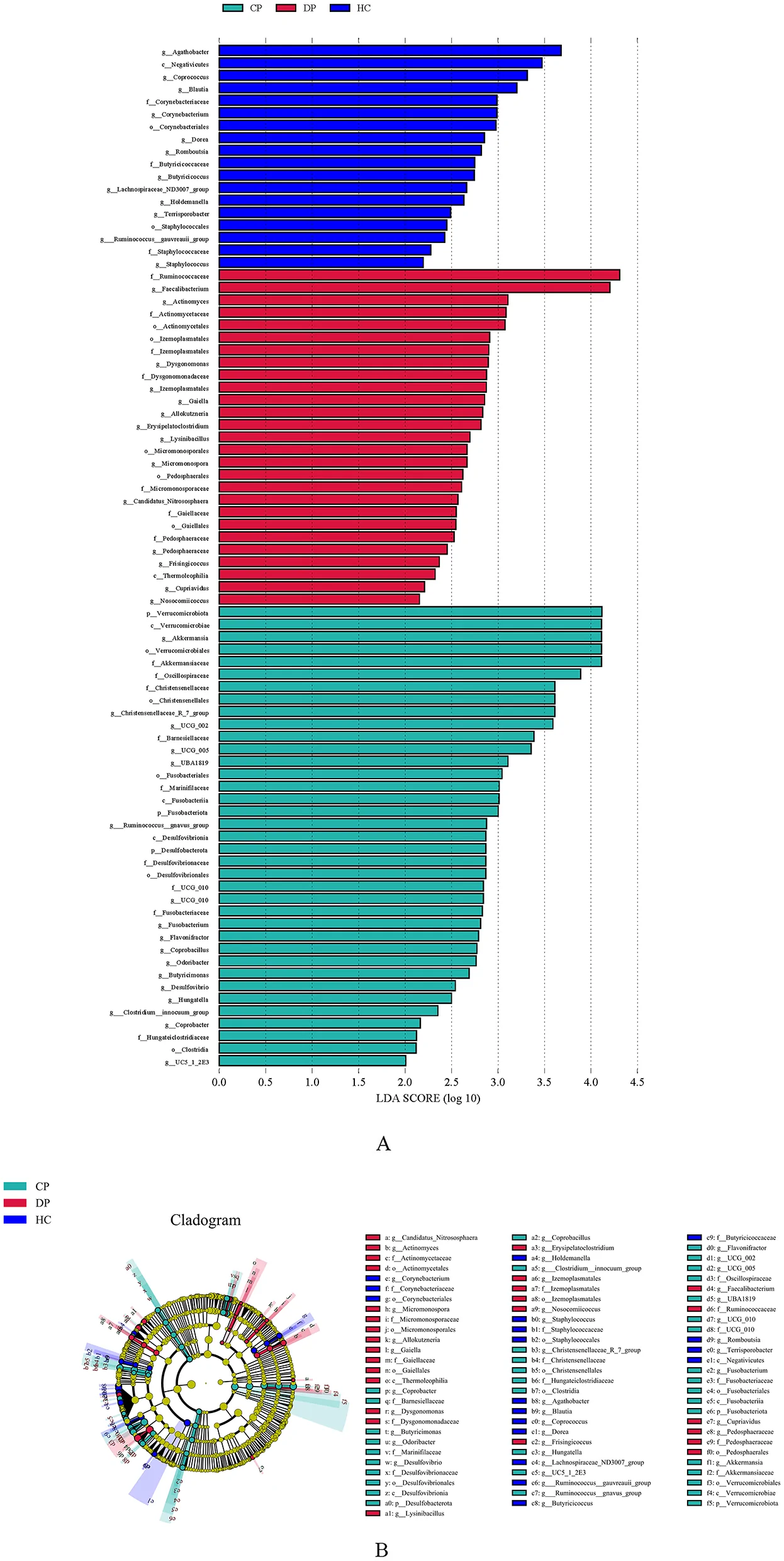
LEfSe analysis of discriminative microbial taxa across the CP, DP, and HC groups. **(A)** LDA score bar chart showing taxa enriched in each group using an LDA threshold > 3.5 and *P* < 0.05. **(B)** Cladogram showing the phylogenetic distribution of enriched taxa across taxonomic levels.

LEfSe analysis was performed to identify microbial taxa contributing to group discrimination across multiple taxonomic levels (Figures 4A, B). The CP group showed the broadest enrichment pattern, dominated by Verrucomicrobiota-associated lineages, including class Verrucomicrobiae, order Verrucomicrobiales, family Akkermansiaceae, and genus *Akkermansia*. Additional CP-enriched taxa included Christensenellaceae R-7 group, UCG-002, UCG-005, and selected taxa within Desulfobacterota and Fusobacteriota (LDA score > 3.5, *P* < 0.05). These findings were consistent with the genus-level results showing a prominent CP-associated microbial pattern involving mucin-associated and inflammation-related taxa.

The HC group was enriched in several health-associated taxa, including *Agathobacter, Coprococcus, Blautia, Dorea, Romboutsia*, and *Butyricicoccus*, as well as class Negativicutes and family Butyricicoccaceae. This enrichment pattern further supported the relative depletion of selected health-associated and SCFA-producing taxa in the disease groups.

The DP group showed a more selective LEfSe profile, mainly involving Ruminococcaceae-related taxa and *Faecalibacterium* (LDA score > 3.5, *P* < 0.05). Together with the genus-level analyses, these findings suggest that DP-associated microbial alterations were more focused than the CP-associated pattern and involved selected Firmicutes-related taxa. The cladogram further illustrated the phylogenetic distribution of differentially enriched taxa, with CP-enriched taxa clustering mainly within Verrucomicrobiota and Desulfobacterota, DP-enriched taxa within Firmicutes, and HC-enriched taxa distributed across health-associated Firmicutes lineages (Figure 4B).

### 3.6 Exploratory random forest classification and microbial feature prioritization

**Figure 5:**
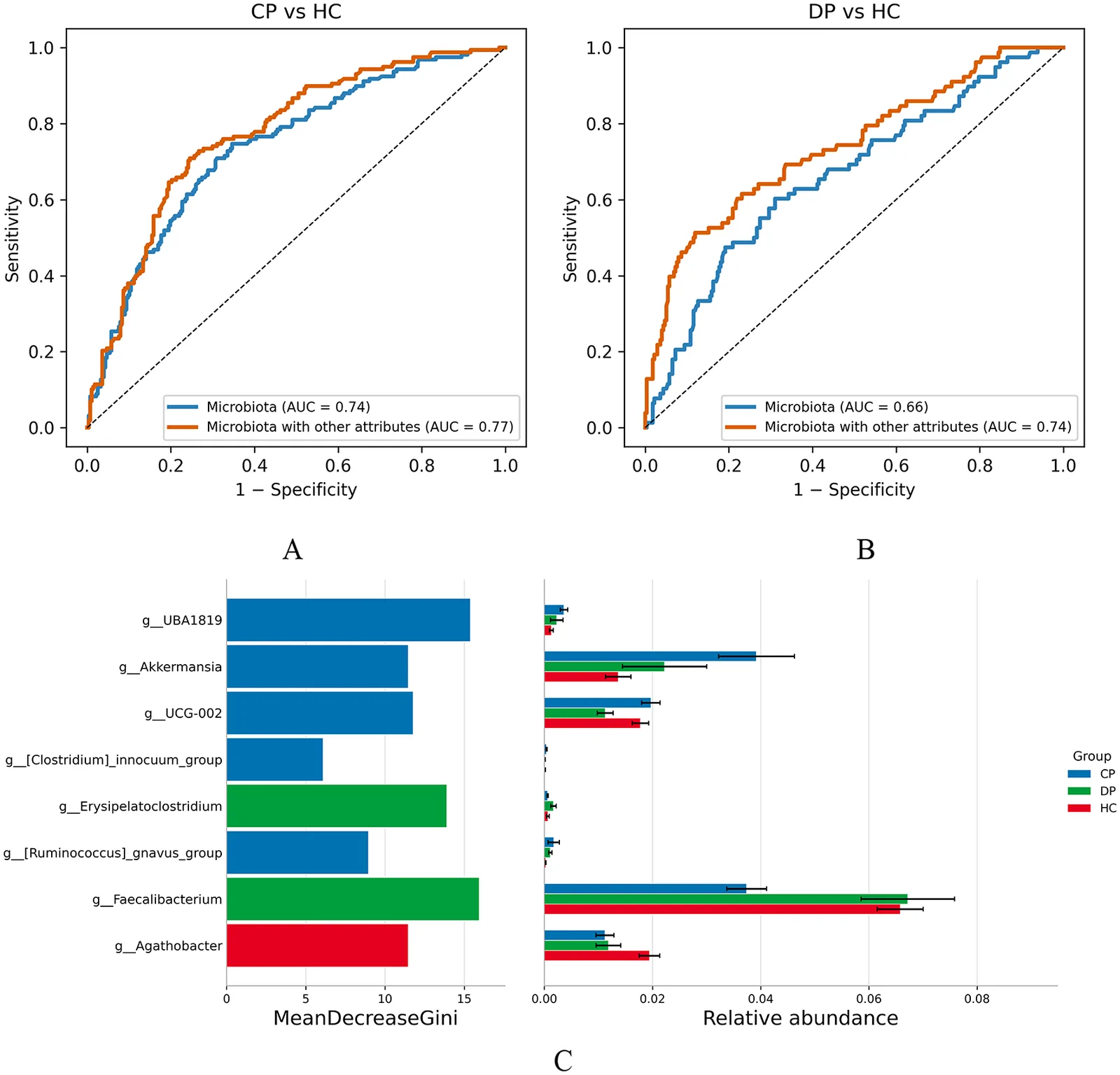
Random forest classification performance and representative microbial feature ranking across the CP, DP, and HC groups. **(A)** ROC curve showing the cross-validated discriminative performance of the pairwise random forest model for CP vs. HC, with the corresponding AUC shown in the panel. **(B)** ROC curve showing the cross-validated discriminative performance of the pairwise random forest model for DP vs. HC, with the corresponding AUC shown in the panel. **(C)** Representative microbial features prioritized by variable importance in the three-class random forest model incorporating genus-level microbial abundance features, age, sex, and BMI. For biological interpretability, only microbial features are displayed. The left panel shows variable importance measured by mean decrease in Gini impurity, and the right panel shows the relative abundance of the corresponding genera across the CP, DP, and HC groups.

Random forest models were constructed to evaluate whether genus-level microbial profiles contained group-related discriminatory information and to prioritize microbial features contributing to group classification. Pairwise random forest models were used to evaluate cross-validated discriminative performance using ROC/AUC (Figure 5A, B). These models showed discrimination for CP vs. HC (AUC = 0.694), DP vs. HC (AUC = 0.602), and CP vs. DP (AUC = 0.615), with the strongest performance observed for CP vs. HC. In complementary analyses, a three-class random forest model incorporating genus-level microbial abundance features, age, sex, and BMI was used to prioritize microbial features contributing to overall discrimination among CP, DP, and HC.

Feature-prioritization analysis highlighted microbial taxa broadly consistent with the differential abundance results. Representative prioritized microbial features included UBA1819, *Akkermansia*, UCG-002, [*Clostridium*]_innocuum_group, *Erysipelatoclostridium*, [*Ruminococcus*]_gnavus_group, *Faecalibacterium*, and *Agathobacter* (Figure 5C). These features captured CP-associated, DP-associated, and HC-associated microbial patterns and provided complementary support for the genus-level differential abundance analyses.

## 4 Discussion

In this study, we characterized genus-level gut microbiota profiles in older adults with constipation, depression, and healthy controls using 16S rRNA sequencing data from the American Gut Project. We found comparable alpha diversity across groups, but beta-diversity and genus-level analyses revealed detectable differences in microbial community composition, with the most consistent signal observed between CP and HC. DP-related alterations were mainly reflected by selected genus-level changes rather than broad community-level separation. These findings are clinically relevant because constipation is common in older adults and depression frequently coexists with gastrointestinal symptoms, both of which may impair quality of life and long-term health outcomes (Suares and Ford, 2011; Lee et al., 2025; Ren et al., 2025). Aging is also accompanied by gut microbiota remodeling, increased inter-individual variability, reduced ecological resilience, and low-grade inflammatory activity, which may influence host-microbiota interactions (Claesson et al., 2012; Xu et al., 2021; Bradley and Haran, 2024). Previous studies have linked chronic constipation and depressive disorder to altered gut microbial composition, although reported taxa differ across cohorts and analytical methods (Parthasarathy et al., 2016, 2017; Gao et al., 2023). Therefore, our findings extend previous work by showing that, in an older-adult population, constipation and depression may share selected genus-level microbiota alterations while also retaining condition-associated microbial features.

After accounting for baseline differences in age, sex, and BMI, the CP group showed the most consistent microbial alteration pattern, especially in the CP-HC comparison. This pattern was characterized by enrichment of *Akkermansia* and depletion of health-associated butyrate-producing taxa such as *Faecalibacterium* and *Agathobacter*. *Akkermansia* is a mucin-degrading bacterium that contributes to mucosal homeostasis under physiological conditions, but its expansion may also reflect altered mucus-layer dynamics in specific disease contexts (Derrien et al., 2017; Cao et al., 2017; Wang et al., 2020; Aja et al., 2025). In constipation, prolonged intestinal transit may change mucin availability, luminal pH, fermentation patterns, and epithelial-microbial interactions, thereby favoring selected mucin-associated taxa (Cao et al., 2017). Therefore, the enrichment of *Akkermansia* in CP should not be interpreted simply as beneficial or harmful, but rather as a marker of altered mucosal ecological conditions associated with constipation.

The depletion of *Faecalibacterium* and *Agathobacter* provides a plausible connection between microbial composition and constipation-related physiology. *Faecalibacterium*, particularly *Faecalibacterium prausnitzii*, is a major butyrate-producing and anti-inflammatory commensal in the human gut (Sokol et al., 2008; Miquel et al., 2013; Quévrain et al., 2016; Ferreira-Halder et al., 2017; Breyner et al., 2017; Rabiei et al., 2019). Butyrate supports epithelial energy metabolism, enhances tight-junction integrity, modulates mucosal immune responses, and contributes to intestinal barrier function (Peng et al., 2009; Canfora et al., 2015; Quévrain et al., 2016; Breyner et al., 2017; Pérez-Reytor et al., 2021). SCFAs can also influence enteric neurons, intestinal secretion, smooth muscle activity, and gastrointestinal motility (Wegener et al., 2006; Nøhr et al., 2013; Jin et al., 2021). In addition, microbial regulation of bile acid metabolism may affect intestinal transit through bile acid receptors such as TGR5 (Duboc et al., 2014; Theriot et al., 2016; Jia et al., 2018). Thus, depletion of butyrate-producing genera in CP may be relevant to constipation through combined effects on barrier function, mucosal inflammation, bile acid signaling, and motility regulation.

DP-related microbial alterations were less extensive at the global community level but remained detectable at the genus level. Across adjusted and sensitivity analyses, DP was associated with enrichment of *Erysipelatoclostridium* and [*Ruminococcus*]_gnavus_group, together with depletion of UCG-002 and selected health-associated genera. Previous depression microbiome studies have reported altered gut microbial profiles in depressive disorder, although the specific taxa vary across cohorts, populations, phenotype definitions, medication exposure, and analytical methods (Liang et al., 2023). The enrichment of [*Ruminococcus*]_gnavus_group may be relevant because this taxon has been linked to inflammatory and mucin-associated dysbiosis in several disease contexts. Reduced abundance of SCFA-associated taxa may also be biologically relevant, as microbial SCFAs have been implicated in immune regulation, microglial activation, and neuroinflammatory processes (Caetano-Silva et al., 2023; Shen et al., 2024; Lv et al., 2024; Cheng et al., 2024). More broadly, SCFAs may contribute to microbiota-gut-brain communication through effects on epithelial barrier function, enteroendocrine signaling, and neural-immune pathways (Hong et al., 2012; Dalile et al., 2019; Silva et al., 2020; Mansuy-Aubert and Ravussin, 2023; Khantakova et al., 2023; Sun et al., 2024).

The overlap between CP and DP appears to lie less in complete disease-specific clustering and more in shared changes among selected genus-level microbial features. Both conditions involved alterations in taxa previously associated with SCFA production, mucin utilization, inflammatory activity, or gut ecosystem stability. These findings suggest that constipation and depression may not share a single uniform microbial signature, but may converge on related ecological and metabolic disturbances within the gut microbiota. SCFA-related alterations are particularly relevant because SCFAs can influence epithelial barrier integrity, mucosal immune regulation, enteroendocrine signaling, and enteric nervous system activity (Nøhr et al., 2013; Canfora et al., 2015). In parallel, microbiota-related changes in tryptophan-serotonin metabolism, bile acid signaling, neuroimmune activation, and HPA-axis activity may provide additional routes through which gut microbial disturbances communicate with mood- and motility-related pathways (Mayer et al., 2014; O'Mahony et al., 2015; Pinto-Sanchez et al., 2017; Kennedy et al., 2017; Valles-Colomer et al., 2019; Jiang et al., 2022; Jia et al., 2024). From this perspective, constipation and depression may share selected microbiota-related biological pathways while retaining condition-associated microbial features. The present findings therefore support the value of population-level 16S rRNA profiling for identifying candidate microbial signatures that can guide future longitudinal, metagenomic, metabolomic, and mechanistic studies.

## 5 Strengths and limitations

Several limitations should be acknowledged. First, phenotype classification was based on publicly available GMrepo metadata rather than clinician-adjudicated diagnoses, and information on symptom severity was unavailable. Second, although covariate-adjusted analyses were performed to account for differences in sex distribution and BMI among groups, several important host and environmental factors—including diet, physical activity, hormone levels, antidepressant use, laxative use, and recent antibiotic exposure—were not available in the public metadata, and residual confounding cannot be fully excluded. Third, participants meeting criteria for both depression and constipation were excluded from the primary mutually exclusive comparison; therefore, the microbiota profile of comorbid depression and constipation could not be directly assessed. Finally, 16S rRNA sequencing provides limited taxonomic and functional resolution and cannot directly characterize microbial genes, metabolites, inflammatory markers, bile acid profiles, neurotransmitter pathways, or causal mechanisms. Future longitudinal studies with validated phenotypes, detailed medication and dietary records, metagenomic sequencing, metabolomic profiling, and host biomarker measurements are needed. Nevertheless, by analyzing a large publicly available older-adult cohort, this study identifies candidate microbial features associated with constipation and depression and provides a basis for future mechanistic and longitudinal investigations.

## Data Availability

The original contributions presented in the study are included in the article/supplementary material, further inquiries can be directed to the corresponding author.
