# Correction: Shared and condition-associated gut microbiota alterations in older adults with depression and constipation: evidence from the American Gut Project

**DOI:** 10.3389/fmicb.2026.1957781

**Published:** 2026-08-19

**Authors:** Xiang Li, Leyong Ke, Ting Wang, Ziqiu Lei, Feng Tian, Yan Zhang, Xin Liu

**Affiliations:** 1 Department of Gastroenterology, Zigong Fourth People's Hospital, Zigong, China; 2 Department of Plastic Surgery, Zigong Fourth People's Hospital, Zigong, China; 3 Department of Gastroenterology, The Affiliated Hospital of Southwest Medical University, Luzhou, China; 4 Department of Clinical Laboratory, Zigong Fourth People's Hospital, Zigong, China

**Keywords:** 16srRNA, American Gut Project, constipation, depression, microbiota, older adults

Affiliations

^1^Department of Gastroenterology, Zigong Fourth People's Hospital, Zigong, China

^2^Department of Plastic Surgery, Zigong Fourth People's Hospital, Zigong, China

^4^Department of Clinical Laboratory, Zigong Fourth People's Hospital, Zigong, China

were erroneously given as

1. Department of Gastroenterology, The Fourth People's Hospital of Zigong City, Zigong, China.

2. Department of Cosmetic and Plastic Surgery, The Fourth People's Hospital of Zigong City, Zigong, China.

4. Department of Clinical Laboratory, The Fourth People's Hospital of Zigong City, Zigong, China.

The original version of this article has been updated.

